# The Rise of FXR1: Escaping Cellular Senescence in Head and Neck Squamous Cell Carcinoma

**DOI:** 10.1371/journal.pgen.1006344

**Published:** 2016-11-03

**Authors:** Erlinda Fernández, Frédérick A. Mallette

**Affiliations:** 1 Chromatin Structure and Cellular Senescence Research Unit, Maisonneuve-Rosemont Hospital Research Centre, Montréal, Quebec, Canada; 2 Département de Médecine, Université de Montréal, Succ. Centre-Ville, Montréal, Quebec, Canada; McGill University, CANADA

Cellular senescence is a key tumor-suppressing mechanism in response to numerous cellular threats including oxidative stress, telomere loss, and oncogene activation. It is essentially a permanent state of G1 cell cycle arrest in which cells remain viable and metabolically active. Recent studies indicate that senescence plays a pivotal role in suppression of tumorigenesis in vivo [1,2] and is frequently observed in different premalignant tumors such as lung adenomas, neurofibromas, and naevi [3,4]. Aside from its critical role in preventing cancer development, the senescence program also enhances the response to cancer therapy [5].

In order to become cancerous, cells must find ways to inactivate or bypass the senescence response. In fact, the viral oncoproteins E6 and E7 from human papillomavirus (HPV), which inhibit the tumor suppressors p53 and Rb, respectively, inactivate cellular senescence in response to oncogenic stress [6,7]. Thus, infection with HPV, an important risk factor for subsets of head and neck squamous cell carcinoma (HNSCC), could promote tumorigenesis by inhibiting cellular senescence. However, numerous HNSCCs are HPV-independent, thus underscoring the need to identify additional genetic alterations in HNSCC. In the September 2016 issue of *PLOS Genetics*, Majumber et al. reported that the Fragile X-related protein 1 (FXR1), an RNA-binding protein, suppresses the senescence response in two different HPV-negative HNSCC cell lines [8]. This further supports the requirement for bypassing senescence in both HPV-positive and -negative HNSCC and sheds light on the putative role of FXR1 in promoting HNSCC.

## Bypass of Cellular Senescence by FXR1 and Transformation

FXR1 is a member of the Fragile X-related family of RNA-binding proteins, which also includes Fragile X Mental Retardation 1 (FMR1) and FXR2 and is frequently amplified in lung squamous cell carcinoma [9]. Majumber et al. have now revealed significant copy number amplification and mRNA overexpression of FXR1 in HNSCC by interrogating cancer genomics databases. Depletion of FXR1 in HNSCC cell lines caused G1 cell cycle arrest associated with features of cellular senescence, including senescence-associated β-galactosidase activity [10] and DNA damage foci [11]. The cellular response triggered by FXR1 depletion stimulated p53 levels and activity as indicated by concomitant elevation of p21, a classical p53-target gene mediating senescence [12]. Using a p53-mutated HNSCC cell line, the authors demonstrated p53-dependent up-regulation of p21 upon FXR1 knockdown. In addition, they confirmed the previously-described ability of FXR1 to directly destabilize p21 mRNA [13] in HNSCC, thereby revealing that FXR1 engages complementary p53-dependent and -independent mechanisms to control p21 levels in HNSCC. Further mining of genomics databases, as well as FISH analysis of tissue microarray and RNA quantification, revealed amplification of the telomerase RNA component TERC in HNSCC. The authors demonstrated that FXR1 is responsible for stabilizing TERC RNA and identified a G-rich region of TERC RNA that physically interacts with FXR1. Ultimately, the decreased TERC levels upon silencing of FXR1 interfered with telomerase activity, thus suggesting a role for FXR1 in acquisition of cell immortality, a critical prerequisite of neoplastic transformation [14]. The authors also demonstrated that depletion of p21 or ectopic expression of TERC allows bypass of senescence subsequent to loss of FXR1, further supporting their observation regarding FXR1-dependent regulation of p21 and TERC.

**Fig 1:**
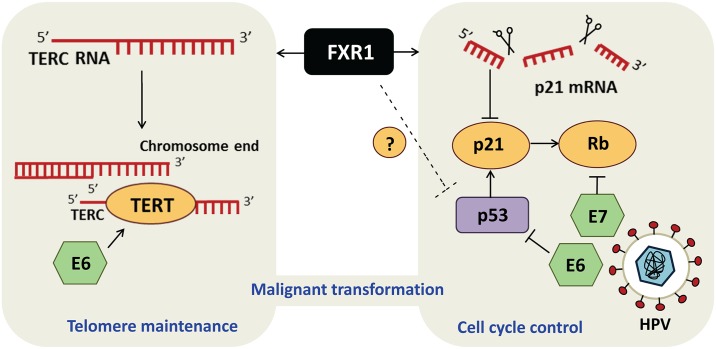
FXR1 engages dual mechanisms to promote malignant transformation in HNSCC. RNA-binding protein FXR1 regulates TERC RNA and p21 mRNA turnover to modulate telomerase activity (left panel) and cell cycle (right panel), respectively, to allow escape from senescence and stimulate transformation. Interestingly, the HPV oncoproteins E6 and E7, which contribute to subtypes of HNSCC, also modulate the p53/p21/Rb tumor suppressor pathways and telomerase activity, like FXR1.

Only a handful of RNA-binding proteins have thus far been described for their role in modulating cellular senescence [15]. Majumber et al. now add FXR1 to this very short list by showing that this factor modulates p21 expression and stabilizes TERC RNA, in turn engaging different mechanisms to counteract cellular senescence and promote transformation (Fig 1). It would now be relevant to investigate whether the functionally related FMR1 and FXR2 also modulate cellular senescence. Also, further investigation of FXR1 amplification during HPV infection would provide additional clues as to whether FXR1 amplification and E6/E7 HPV proteins are mutually exclusive or cooperate to promote HNSCC. In addition, whether the requirement for FXR1 in cancer development is specific to HNSCC, or could apply to other tumour types exhibiting FXR1 amplification such as lung cancer [9], remains to be determined.

## Modulating FXR1 Activity to Treat HNSCC and Facioscapulohumeral Muscular Dystrophy?

The senescence response triggered upon FXR1 depletion has been elegantly described and characterized by Majumber et al. and highlights the potential interest of interfering with the RNA-binding functions of FXR1 to treat oral squamous cell carcinoma (OSCC), the most common oral cancer. Nevertheless, the precise contribution of FXR1 amplification in the complex sequence of events leading to OSCC, as well as the underlying molecular mechanisms, remain to be elucidated. This would be expected to reveal additional therapeutic avenues to limit FXR1 expression in HNSCC.

In addition to its amplification in lung, breast, and ovarian as well as head and neck cancers [8,9], FXR1 was shown to be down-regulated in other human pathologies such as facioscapulohumeral muscular dystrophy, an inherited myopathy [16]. FXR1 plays an important role in myogenesis in both mouse and *Xenopus*. FXR1 knockout mice die shortly after birth and display disruption of the cellular architecture of skeletal and cardiac muscle [17]. Majumber et al. demonstrated that loss of FXR1 not only leads to senescence in cancer cells, but also in normal cells such as mouse embryonic fibroblasts [8]. Considering that cellular senescence limits the muscle stem cell regenerative capacity [18], it is reasonable to envisage that the phenotype observed following FXR1 dysfunction in facioscapulohumeral muscular dystrophy could be caused by concomitant overstimulation of cellular senescence, thus leading to muscle tissue degeneration. This considerably extends the potential reach of the discovery made by Majumber et al.

## Cell Fate Decision by FXR1: Cellular Senescence or Quiescence?

The work of Majumber et al. raises an interesting point regarding the potential contribution of FXR1 to cell fate decisions. Previous reports described a role for this protein in mediating translation during quiescence [19]. The recent discovery by Majumber et al. that FXR1 loss promotes senescence, while FXR1 activity participates in quiescence, might provide a hint about the molecular mechanisms involved in making cell cycle arrest fate decisions between senescence and quiescence.
